# Demystifying Large Language Models for Medicine: A Primer

**Published:** 2024-11-20

**Authors:** Qiao Jin, Nicholas Wan, Robert Leaman, Shubo Tian, Zhizheng Wang, Yifan Yang, Zifeng Wang, Guangzhi Xiong, Po-Ting Lai, Qingqing Zhu, Benjamin Hou, Maame Sarfo-Gyamfi, Gongbo Zhang, Aidan Gilson, Balu Bhasuran, Zhe He, Aidong Zhang, Jimeng Sun, Chunhua Weng, Ronald M. Summers, Qingyu Chen, Yifan Peng, Zhiyong Lu

**Affiliations:** 1 National Library of Medicine (NLM), National Institutes of Health (NIH), Bethesda, MD, USA.; 2 Department of Computer Science, University of Illinois Urbana-Champaign, Champaign, IL, USA.; 3 Department of Computer Science, University of Virginia, Charlottesville, VA, USA.; 4 Department of Biomedical Informatics, Columbia University, New York, NY, USA.; 5 School of Medicine, Yale University, New Haven, CT, USA.; 6 School of Information, Florida State University, Tallahassee, FL, USA.; 7 Department of Radiology and Imaging Sciences, NIH Clinical Center, Bethesda, MD, USA.; 8 Department of Population Health Sciences, Weill Cornell Medicine, New York, NY, USA.

## Abstract

Large language models (LLMs) represent a transformative class of AI tools capable of revolutionizing various aspects of healthcare by generating human-like responses across diverse contexts and adapting to novel tasks following human instructions. Their potential application spans a broad range of medical tasks, such as clinical documentation, matching patients to clinical trials, and answering medical questions. In this primer paper, we propose an actionable guideline to help healthcare professionals more efficiently utilize LLMs in their work, along with a set of best practices. This approach consists of several main phases, including formulating the task, choosing LLMs, prompt engineering, fine-tuning, and deployment. We start with the discussion of critical considerations in identifying healthcare tasks that align with the core capabilities of LLMs and selecting models based on the selected task and data, performance requirements, and model interface. We then review the strategies, such as prompt engineering and fine-tuning, to adapt standard LLMs to specialized medical tasks. Deployment considerations, including regulatory compliance, ethical guidelines, and continuous monitoring for fairness and bias, are also discussed. By providing a structured step-by-step methodology, this tutorial aims to equip healthcare professionals with the tools necessary to effectively integrate LLMs into clinical practice, ensuring that these powerful technologies are applied in a safe, reliable, and impactful manner.

## Introduction

Large language models (LLMs), exemplified by GPT-4^1^, Claude 3^2^, Gemini 1.5^3^, and Llama 3^4^, are artificial intelligence (AI) models that can generate human-like responses under various conversational contexts and adapt to novel tasks by following human instructions^5,6^. They have shown great promise in diverse biomedical and healthcare applications^7,8,9,10,11^, such as question answering^12,13,14,15^, clinical trial matching^16,17,18^, clinical documentation^19,20,21^, and multi-modal comprehension^22,23^.

**Figure 1.**
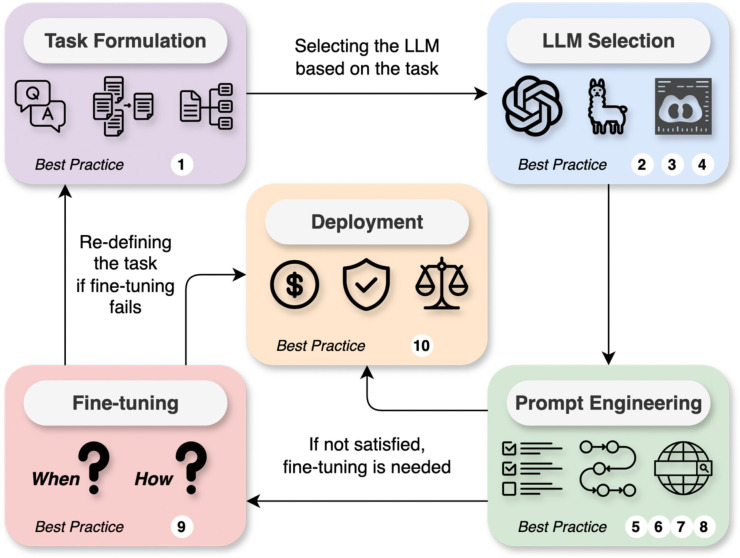
Overview of the proposed systematic approach to utilizing large language models in medicine. Users need to first formulate the medical task and select the LLM accordingly. Then, users can try different prompt engineering approaches with the selected LLM to solve the task. If the results are not satisfying, users can fine-tune the LLMs. After the method development, users also need to consider various factors at the deployment stage. Corresponding best practices in Box 1 are also listed in each phase.

Despite the accelerated progress of LLMs in patient care and clinical practice, biomedical and health sciences research, and education^8,9,24^, there is a noticeable lack of practical, actionable guidelines for their application from bench to bedside and beyond. A lot of current use of LLMs, such as ad-hoc prompting with ChatGPT, is far from sufficient in medical tasks^25,26^. This gap can lead to both underutilization and misapplication of these technologies, potentially affecting patient outcomes. Our work aims to close this gap by providing a detailed, structured framework to guide the utilization and integration of LLMs into medical workflows (Figure 1). Specifically, this framework consists of task formulation, model selection, prompt engineering, fine-tuning, and deployment considerations. We further provide a set of best practices (Box 1) while supporting adherence to ethical use, evaluation metrics, and compliance standards. The tutorial code that contains step-by-step instructions is publicly available at https://github.com/ncbi-nlp/LLM-Medicine-Primer. We hope this work will help equip healthcare professionals with the necessary knowledge to effectively leverage LLMs in their practices.

## Task Formulation

Adapting an abstractive medical need into one or more concrete tasks that can be addressed by an LLM requires users to first understand the core capabilities of LLMs, which we classify into five broad categories: (1) knowledge and reasoning, (2) summarization, (3) translation, (4) structurization, and (5) multi-modal data analysis. We recommend beginning by identifying the primary LLM capability relevant to your task. Once the task is formulated, one should aim to collect a diverse set of instances (test cases) that contain input and output data elements for development (Box 1, Best Practice 1). We recommend collecting about 100 test instances, following several evaluation studies of LLMs in medicine^14,20,27^.

### Knowledge and reasoning

LLMs can use the medical knowledge encoded in their parameters to perform domain-specific reasoning given different contextual information^10,12,13,14,28,29^. This capability can enable a variety of medical applications, such as answering medical questions^30^, clinical decision support^31^, and matching patients to clinical trials^16,17,18^. Task instances usually include question (input), explanation (reasoning and context output), and short answer (output). When formulating a reasoning task, users can quickly evaluate short answers (e.g., yes/no) between LLM predictions and ground truth and proceed to in-depth analyses of explanations if short answers are satisfying^27^.

### Summarization

LLMs can summarize complex documents into concise paragraphs or sentences. Summarization tasks in biomedicine primarily fall into two categories: (1) summarizing long clinical notes into shorter texts, such as generating the progress notes and discharge summaries^20,21^; (2) summarizing medical literature for evidence synthesis, such as generating the systematic reviews given a list of clinical studies^32,33,34^. These labor-intensive tasks can be potentially streamlined by LLMs^19,35^. For a summarization task, instances include instruction (input), original text (input), and summarized text (output). Users may leverage metrics like BLEU^36^, ROUGE^37^, and BERTScore^38^ to compare the generated texts and the reference summaries. However, it should be noted that automatic metrics do not always correlate well with the gold-standard human judgments^39^.

### Translation

LLMs also have the capability to translate text, not only between different languages but also between writing styles appropriate for different audiences. This ability can enable applications such as sharing medical knowledge across different language demographics^40^, supporting medical education^41,42^, and facilitating communication with patients^43^. A translation task, similar to a summarization task, includes instances made of instruction (input), source text (input), and target text (output) and is evaluated similarly to a summarization task.

### Structurization

LLMs can be leveraged to convert free-text input into structured outputs such as a list of key-value pairs. Medical structurization problems include classifying an input text into controlled vocabularies like the diagnosis-related groups^44^ and extracting biomedical concepts as well as their relationships (e.g., variant-*causing*-disease) from unstructured text^45^. Structurization instances include task instruction (input), source text (input), and a list of extracted concepts and relations in structured forms (output). The evaluations are made to match the output with the reference answers. As such, the performance is often typically measured by precision, recall, and F1 score, etc.

### Multi-modality

Multi-modal LLMs like GPT-4 can analyze and integrate diverse data types such as text, images, audio, and even genomic information, potentially serving as a generalist medical AI^22^. For example, these models can support clinical tasks, such as generating radiology reports and guiding clinical decisions^46,47,48^ based on real-world multimodal patient data. The multi-modal task instances and evaluations are similar to those of the knowledge and reasoning tasks, except that the input question typically contains data in multiple modalities (e.g., past medical history in EHR with current imaging results).

## Large Language Model Selection

**Figure 3.**
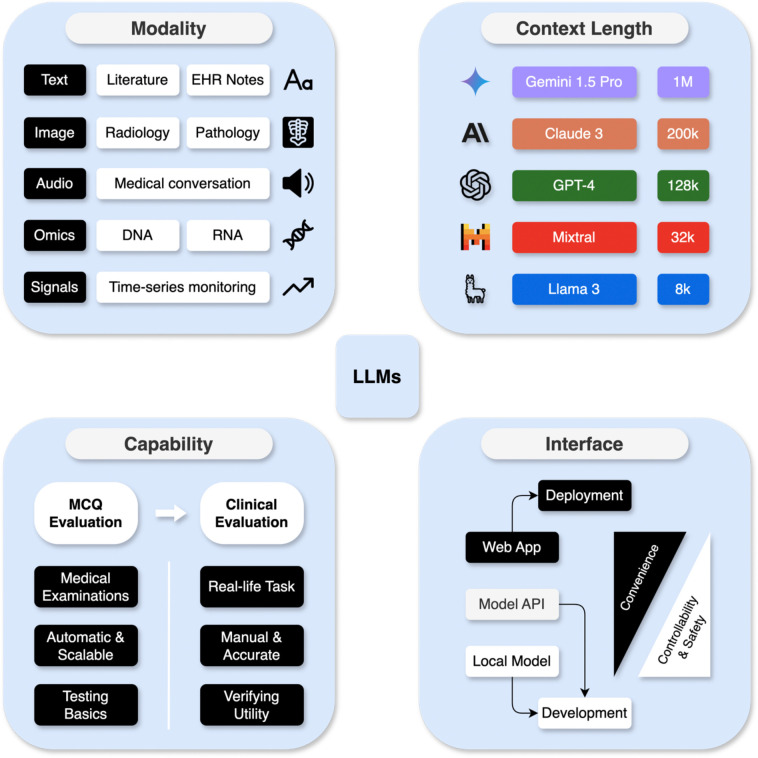
Considerations of choosing the LLMs. Users need to choose LLMs that can handle the modality and context length of the selected task. They also need to understand the capabilities of LLMs in the medical task. The gold standards come from manual evaluation of the model’s behavior on real clinical tasks, but this is expensive and time-consuming. Models might be screened for basic medical capabilities with automatic evaluations on medical examinations first, and only models that pass the medical examinations are suitable for clinical evaluation. During the development phase, users need to use the model APIs and (or) local models for better controllability and safety features. Web applications such as online chatbots are suitable for deployment to reach more users. EHR: Electronic medical records. MCQ: multiple-choice questions.

Users should choose an appropriate LLM based on their task characteristics. There are a wide variety of LLMs, including proprietary models such as GPT-4^1^, Gemini^3^, Claude^2^, and open-source models such as Llama^4^ and Mistral^49^. They can range in size from several billion parameters (e.g., Llama3.1–8B) to hundreds of billion parameters (e.g., Llama3.1–405B). Typically, larger models exhibit more proficient responses^50^. Some models are trained for more general applications, while others such as PMC-LlaMA^51^ and MEDITRON^52^ are fine-tuned for specific domains or applications. When choosing the LLM, users should consider three key factors in their needs: Task and Data, Performance Requirements, and Model Interface. Figure 3 shows an overview of the LLM selection considerations discussed in this section, and Table 1 shows the characteristics of commonly used LLMs.

### Task and Data

The first and most critical factor to consider is the nature of the data the user is working with and the specific task to perform. Ensuring that the chosen model aligns with the data type and task requirements is foundational to successful LLM implementation. When working with sensitive patient data, it is important to ensure privacy and compliance with regulations such as Health Insurance Privacy and Accountability Act (HIPAA). Proprietary models accessed through APIs like OpenAI’s GPT-4 are typically not HIPAA-compliant, so they should not be used for patient data. In contrast, certain cloud service providers such as Azure and Anthropic provide HIPAA-compliant access to LLMs, which could be potentially used for sensitive data. Alternatively, local models such as Llama or Mistral can be used for enhanced control over privacy and security when processing sensitive medical information.

The diversity of healthcare tasks necessitates processing various data modalities in addition to free texts. Radiology and pathology applications, for example, may require models that can interpret and generate insights from 2D or 3D medical images, which requires models beyond text-only LLMs like GPT-3.5 and Llama 3. Medical conversations produce audio data, which can be transcribed into text for processing by text-based LLMs. Genomics data, including DNA sequences and RNA expressions, require the knowledge of omics data interpretation^58^. Similarly, time-series data, such as monitoring vital signs, need models that can analyze long temporal patterns in structured EHR. While both genomics and time-series data can be represented as free-text, it remains unclear whether general LLMs can effectively handle such inputs without further training or adaptation. Users should determine what data modalities are essential to their task and select an LLM that can support such modalities (Box 1, Best Practice 3).

For tasks that involve large inputs, the length of the input data that the model can handle is crucial. Users must understand the length distribution of their datasets and choose an LLM with a context window that can accommodate the input data (Box 1, Best Practice 3). The title and abstract of a PubMed article, for instance, consist of roughly 250 words, or about 300–400 tokens (model input units). As demonstrated in the online tutorial, one token is about 0.8 words as text is often tokenized into subwords and individual characters. Some open-source models like Llama 3 have a limited context window (the longest input prompt it can process) of 8,000 tokens, which is about 20 abstracts. In contrast, long-context LLMs like GPT-4 (128k tokens context window), Claude 3 (200k tokens), and Gemini 1.5 Pro (1M tokens) can process approximately 320, 800, and 2,500 PubMed articles, respectively. Selecting LLMs with appropriate context windows ensures efficient processing of long input, but it should be noted that issues such as lost-in-the-middle^59^ (where the model fails to utilize information in the middle of the prompt) also appear in long-context LLMs.

### Performance Requirements

The model’s medical capabilities are one of the most critical factors to consider when selecting LLMs. Typically, greater capabilities come with larger model sizes which require more resources for development and customization. Conversely, smaller LLMs may not perform as well as their larger counterparts, but they are often more sustainable and less costly. While LLM capabilities vary across different model sizes, capabilities are also affected by the target applications. For example, LLMs are better for tasks that require medical knowledge and clinical reasoning but do not often outperform fine-tuned BERT models^60^ in simpler tasks such as structurization^61^.

There are two main approaches to evaluating the medical capabilities of LLMs: multi-choice question (MCQ) evaluation and clinical evaluation. Medical examination and question-answering tasks, such as MedQA-USMLE^53^, PubMedQA^62^, MedMCQA^63^, have been commonly used to evaluate the knowledge and reasoning capabilities of LLMs^14^. These benchmarks should only be used to filter out models that cannot meet basic performance standards. However, higher scores on these datasets do not necessarily translate to better clinical utility, as there are no choices provided in real-life applications. After a model passes initial screening via MCQ evaluations, it must be further assessed for clinical utility. This involves rigorous testing, such as randomized controlled trials, to ensure the model’s outputs are trustworthy and beneficial in real-world healthcare settings.

In summary, MCQ evaluation can be used to screen LLMs for basic medical capability in a scalable way, while clinical evaluation can provide a gold standard relevant to patient care at the cost of greater human effort. When selecting LLMs, we recommend that users choose LLMs based on clinically evaluated results. However, clinical evaluation is challenging and may not be readily available. In such scenarios, users should consider using medical examination results to guide the selection of LLMs for further clinical evaluations. (Box 1, Best Practice 4)

### Model Interface

Once the LLM(s) has been chosen, the users also need to select a point of access to the LLMs based on their needs. Broadly, there are three ways to access LLMs: web applications, model application programming interfaces (APIs), and locally hosted implementations. Web applications, such as ChatGPT, are inexpensive and easy to use compared to APIs and local models; however, they do not provide interfaces that allow flexible control of model behavior and large-scale evaluations. In addition, most LLM web applications do not have clear compliance with standards such as HIPAA, which further raises security issues when dealing with sensitive patient data. Consequently, we do not recommend using web applications during the development phase. In contrast, model APIs are controlled points of access via the web that provide an interface to proprietary models such as PaLM and open-source models like Llama 3. They are typically easier to use than implementing local LLMs, and some of the model APIs provide HIPAA-compliant services. Lastly, locally hosted LLM implementations are often derived from open-source models. In general, local models provide greater control and privacy^7^. For instance, accessing specific parameters and getting the raw prediction of the next token distribution is possible in a local model but often not with a model API or via Web applications. Overall, while cutting-edge proprietary LLMs often deliver better performance in general tasks, users may have less control over customization, privacy, and safety. Conversely, open-source LLMs allow for greater customization and security but demand more GPU resources and technical expertise for both the development and the deployment phases.

Given the variety of LLMs available for medical applications, LLM selection requires careful considerations. While the supported data modalities and context windows are hard constraints, users must navigate trade-offs between model interface and medical capability based on their specific needs. For example, users may want to select proprietary LLMs of larger size when general capability has high priority. In contrast, users may opt to use local models when customizability is a concern. Ultimately, users must examine their task and select one (or more) LLM that satisfies their priorities.

## Prompt engineering

**Figure 4.**
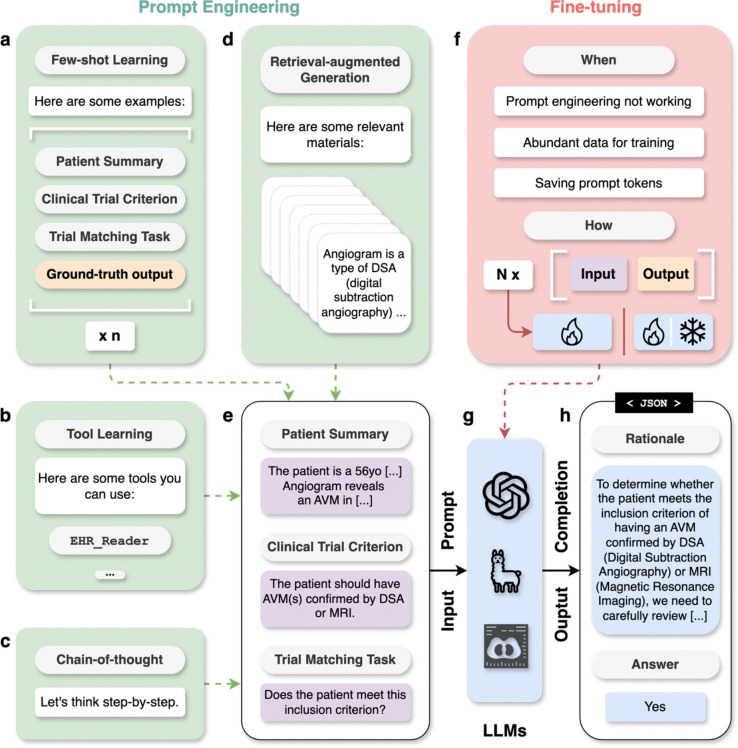
An overview of prompt engineering and fine-tuning techniques. **a**, Task examples are shown to the model in few-shot learning (FSL). **b**, Tool learning provides the model with access to external tools like database utilities. **c**, Chain-of-thought (CoT) prompting instructs the model to generate step-by-step rationale. **d**, Retrieval-augmented generation (RAG) provides relevant materials to solve the task. **e**, The patient-to-trial matching task where the patient summary and the clinical trial eligibility criterion are given. **f**, An overview of fine-tuning, including when and how to perform it. **g**, The inputs to LLMs are known as “prompts”, and their outputs are “completions”. **h**, An example output of LLMs that contain the CoT rationale as well as the short answer, organized in the JSON format. n denotes the number of shots for few-shot learning, and N denotes the number of instances for fine-tuning.

Harnessing LLM capabilities for application to a specific task requires careful consideration of the prompt (input content) given to the model. Prompt engineering is the process of designing and optimizing prompts to effectively guide LLMs in generating accurate and coherent responses^64^. Prompts can vary from one simple instruction to many documents retrieved by a search system, allowing users to elicit a variety of behaviors without the need to modify the parameters of LLM. In general, more complex tasks typically require more sophisticated prompts. Figure 4 shows a concrete task example of clinical trial matching, where a simple prompt that merely describes the patient and lists the clinical trial criteria (shown in Fig. 4e) might not work well. As such, prompt engineering and fine-tuning methods should be used. Table 2 shows resource requirements, advantages, disadvantages, and use cases of different approaches. Some detailed case studies are also listed in Table 3.

### Few-shot learning (FSL)

As shown in Fig. 4a, FSL includes a few examples (i.e., “shots”) within the prompt to better specify the task for the model^5^. Each demonstration example should include both the input and desired output. In the case of patient-to-trial matching, the input contains patient information, clinical trial criterion, and the task instruction; the output contains the rationale and the criterion-level eligibility label. Zero, one, and more than one examples (e.g., five) are respectively denoted as zero-shot, one-shot, and few-shot learning and are the most commonly used in practice. The examples should be as representative and diverse as possible. For example, demonstrations of all potential labels (e.g., disease sub-types) should be shown for a classification task. Another useful approach to few-shot learning is to generate examples dynamically, based on semantic similarity to the instances being predicted^44^. We recommend starting from zero-shot learning and incrementally adding examples to increase performance or deal with edge cases (Box 1, Best Practice 5).

### Chain-of-thought (CoT) prompting

As shown in Fig. 4c, CoT prompting involves designing prompts that lead the model through a step-by-step reasoning process^69^. For example, providing the explanation of the patient-criterion relation helps the users efficiently verify the LLM-predicted eligibility labels. One can simply add “Let’s think step-by-step” to the end of the input for CoT prompting. This technique is particularly useful in complex medical decision-making tasks, where an explanation of reasoning can improve model performance and aid clinicians in understanding and verifying AI-generated advice. We recommend using CoT prompting as the default as it improves the explainability of AI responses and potentially the performance as well (Box 1, Best Practice 6).

### Retrieval-augmented generation (RAG)

In RAG (Fig. 4d), a search engine retrieves relevant documents, such as scientific articles, to be included in the prompt, allowing the model to better solve knowledge-intensive tasks such as answering questions^70^ (Box 1, Best Practice 7). By grounding the LLMs to respond based on the provided relevant textual snippets, RAG can potentially reduce hallucinations^71^ (the generation of incorrect information) and improve upon outdated knowledge encoded in large language models. For example, LLMs can get access to the definition of certain medical concepts with RAG to better classify the patient eligibility in the application of patient-to-trial matching. We recommend using high-quality domain-specific corpora, such as systematic reviews, medical textbooks, and clinical guidelines, for RAG systems in medicine^54^.

### Tool learning

Certain medical tasks require the use of domain-specific tools such as database utilities or medical calculators. If these tools are implemented as application programming interfaces (APIs), LLMs can utilize them through a function calling mechanism (Box 1, Best Practice 7). In the example case of clinical trial matching, LLMs can be provided with tools for reading raw electronic health records to capture detailed information that might be missing from a patient summary (shown in Fig. 4c).

### Setting the temperature

Besides the prompt, LLMs also require a temperature parameter that controls the amount of randomness when generating the response. Lower temperatures result in a more consistent output, while higher temperatures lead to more creative responses. Temperature can usually be set via API or local parameterization, but usually not via Web app. We recommend that users start with a temperature of 0 to get deterministic results for reproducibility and only consider increasing the temperature to get diverse responses for purposes such as ensembling^72^ (Box 1, Best Practice 8).

### Additional considerations

There are several additional aspects that users should consider during prompt engineering, such as approaches for multi-modal data types and formatting the output. Effectively integrating various data types and crafting precise prompts is crucial for maximizing the utility of models like GPT-4. For example, single cell RNA sequencing data can be transformed into detailed textual prompts that include gene markers and expression levels^73^. Similarly, biosensor monitoring signals can also be transformed into texts to enable the generation of personal health insights and exercise recommendations^74^. These prompts help the model to accurately perform multi-modal data analysis. Users should also consider the output format during prompt engineering, with the primary consideration being the difficulty of automatically parsing the response output. We therefore recommend instructing LLMs to generate a structured output, such as a JSON dictionary, to allow the result to be easily parsed into different answer sections (Box 1, Best Practice 8).

### Fine-tuning

Although LLMs can solve many tasks using prompt engineering without explicit model modification, there are at least three situations where fine-tuning may be considered: (1) prompt engineering techniques like few-shot learning and RAG cannot sufficiently improve results, (2) high-quality training data is readily available in large scale, (3) the working prompt is too long to be feasible in terms of cost. (Box 1, Best Practice 9).

LLMs can be fully or partially fine-tuned. Full model fine-tuning updates all the parameters of an LLM, while parameter-efficient fine-tuning (PEFT) methods^83,84,85,86^, such as Low-Rank Adaptation (LoRA^83^), update a subset of LLM parameters or add additional trainable weights to the LLM. In general, smaller and more specific data is suitable for PEFT to prevent overfitting^87^, while larger and more diverse data is suitable for full-scale fine-tuning to better train the model^75^. For example, Med-PaLM 2^28^ used a diverse set of instances spanning medical exams, consumer health information, and medical research. The model utilizes both full fine-tuning and a novel method known as ensemble refinement, achieving high results on several benchmarks. PEFT greatly reduces the hardware requirements for fine-tuning. By using a small set of trainable parameters, quantized LoRA (QLoRA)^84^ uses quantization and adapter methods^84,88^ that reduce fine-tuning memory requirements (e.g., from over 780GB to 48GB) while maintaining fixed model parameters. PEFT approaches are often competitive with full model fine-tuning methods and even outperform them in low-data environments. For example, Van Veen *et al* used QLoRA to fine-tune LLMs for clinical text summarization with thousands of training instances and only one NVIDIA Quadro RTX 8000 GPU^89^.

In summary, we suggest performing full or partial fine-tuning depending on computational resources and dataset features. In addition to open-source LLMs, some proprietary LLMs, such as GPT-4, can also be fine-tuned via file upload to their web applications. However, the implementation details of these fine-tuning approaches are unknown to the public and might raise concerns about transparency and privacy. Similar to prompt engineering approaches, fine-tuned models also need to be evaluated on an independent test set to verify the performance improvement of training.

## Deployment considerations

### Regulatory compliance

Deploying LLMs in the biomedical domain requires adherence to privacy standards such as HIPAA and the General Data Protection Regulation (GDPR^90^) to protect patient data. When utilizing proprietary LLMs, it is essential to ensure that the platforms are HIPAA-compliant. Alternatively, processing data locally using an open-source model can enhance data safety^7^. For example, while the OpenAI API is not currently compliant with HIPAA, Azure services provide HIPAA-compliant access to OpenAI’s models. Similarly, Anthropic provides HIPAA-certified API hosting for its Claude models. AI algorithms like LLMs are potentially regulated as medical devices, especially when used in clinical settings or for decision support. This adds an additional layer of regulatory scrutiny, requiring compliance with relevant standards such as those established by the FDA or other regulatory bodies overseeing medical device software. In the end, users must maintain the ethical and legal integrity of their deployment by carefully selecting protocols that align with compliance requirements and clinical standards (Box 1, Best Practice 10).

### Equity and fairness

Users should evaluate potential biases in LLM’s training data and algorithms to ensure fair and equitable outcomes^91^. Prior work has shown that even the most successful proprietary models can exhibit racial bias. Studies have shown that, when presented with identical patient profiles differing only by race, LLMs can yield varying predictions for treatment, cost, or outcome. Such differences can result in healthcare disparities during production. Thus, it is necessary to evaluate model fairness before deployment^92,93^. When examining data or algorithms is not viable, such as in the case of many proprietary models, users may still use existing benchmark datasets for evaluation^94,95^. This approach provides an idea of whether the models are fair or biased, and to what extent they exhibit bias.

### Costs

When considering the costs associated with deploying large language models, it is important to distinguish between proprietary and open-source models. Proprietary LLMs require usage fees for each request made to the service provider. In the case of OpenAI’s GPT-4 model, this pay-as-you-go (PAYG) system processes each token at a cost of $0.03 per 1,000 prompt tokens for models with 8k context lengths, with additional charges for completion tokens at $0.06 per 1,000 tokens. For a typical MIMIC-III^91^ discharge note containing around 4,000 tokens, processing would cost approximately $0.12 for prompt tokens, with additional costs depending on the response length generated by the model. Some proprietary model providers also offer customization services such as fine-tuning for an additional fee. Though proprietary models typically offer robust support, this pricing structure can cause delays in customization and updates. Utilizing services in this way does not guarantee access to the most advanced updates and limits customization, as new updates undergo extensive quality checks and alignments before companies release them. In contrast, open-source LLMs involve procurement costs for the necessary hardware, like GPUs, and ongoing costs related to maintenance. While the up-front costs are higher when running the model locally, these can be offset by the lower ongoing costs. In addition, an open-source setup can offer additional benefits like the ability to fine-tune the model to specific applications with protected data and more control over system responsiveness and updates. However, users should consider the potential for increased latency and reduced throughput during periods of high local demand. Local devices running LLMs might not match the speed and response time of large companies hosting LLMs.

### Post-deployment

After the deployment of LLMs in healthcare, continuous monitoring is essential (Box 1, Best Practice 10). Users should ensure that LLM outputs are responsibly used as support tools, not as independent replacements for the judgment of healthcare practitioners. Effective training programs^96^ are crucial to help healthcare professionals understand how to interpret and utilize these outputs while managing potential risks. Additionally, the successful integration of LLMs in medical practices demands active collaboration with patients and local communities. This involves leveraging engagement methods, such as community advisory boards and patient panels, to gather meaningful feedback and perspectives^97^. Such inclusive strategies help tailor LLM applications to the real-world diversity of patient experiences and enhance the effectiveness of these technologies in medical practice.

## Conclusions

Large language models (LLMs) have the potential to revolutionize healthcare by enhancing clinical workflows, decision-making, and patient outcomes. However, their effective integration into medical practice requires a systematic and thoughtful approach. This tutorial provides a comprehensive framework for utilizing LLMs in medicine, emphasizing critical stages such as task formulation, model selection, prompt engineering, and deployment. By following these guidelines, healthcare professionals can maximize the benefits of LLMs while addressing challenges related to regulatory compliance, fairness, and cost. As AI continues to evolve, the careful application of these methods will be essential in ensuring that LLMs are used responsibly and effectively, ultimately improving the quality of care delivered to patients.

## Figures and Tables

**Figure 2.**
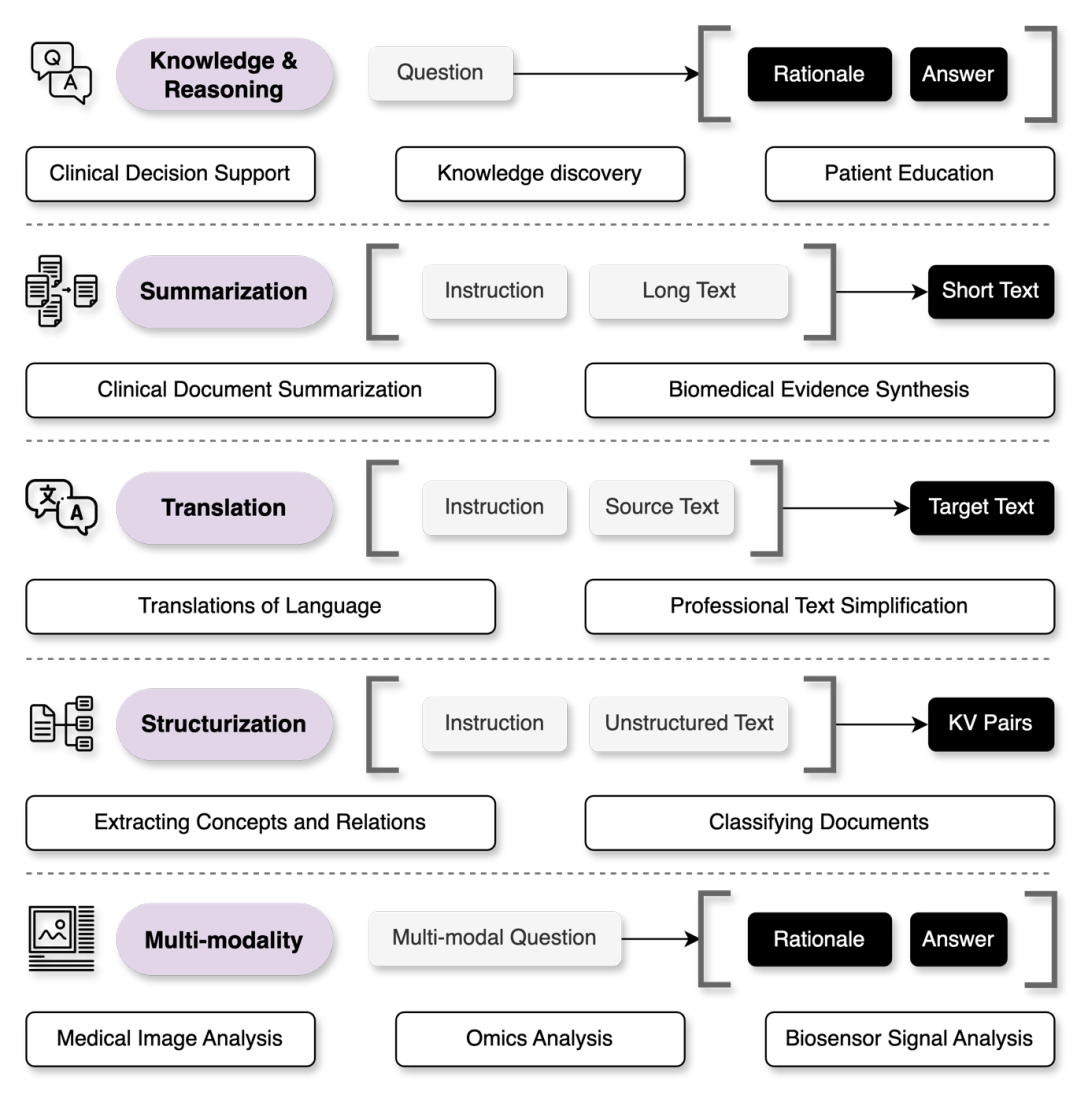
An overview of five common task formulations enabled by LLMs in medicine, with a set of examples. LLMs can answer questions using their domain knowledge and reasoning capabilities. The summarization task shortens long texts into concise summaries. The translation task transforms the source text to the target text in different language styles. The structurization task converts unstructured texts into structured key-value (KV) pairs. LLMs can also be used to support multi-modal data analysis such as interpreting medical images.

**Table 1.** Characteristics of different LLMs, sorted by the best reported MedQA-USMLE^53^ (4 options) score.

LLM	Weights	Size	Interface	Modality	Context	MedQA
**Med-Gemini**	Closed	NA	Web, API	T, I, V, A	1M, 2M	91.1%^6^
**GPT-4**	Closed	NA	Web, API	T, I	8k, 32k, 128k	90.2%^8^
**Med-PaLM 2**	Closed	NA	API	T	8k	86.5%^28^
**Llama 3**	Open	8B, 70B, 405B	API, Local	T	8k	80.9%^54^
**GPT-3.5**	Closed	NA	Web, API	T	4k, 16k	68.7%^55^
**Med-PaLM**	Closed	540B	API	T	8k	67.6%^14^
**Gemini 1.0**	Closed	NA	Web, API	T, I, V	32k	67.0%^56^
**Mixtral**	Open	8x7B	API, Local	T	32k	64.1%^54^
**Mistral**	Open	7B	API, Local	T	8k, 32k	59.6%^57^
**Llama 2**	Open	7B, 70B	API, Local	T	4k	47.8%^54^
**Claude 3**	Closed	NA	Web, API	T, I	200k	N/A

T: text; I: image; V: video; A: audio. The GPT-4 series includes GPT-4, GPT-4-turbo, and GPT-4o. The GPT-3.5 series includes Codex and GPT-3.5-turbo.

**Table 2.** Characteristics of different approaches to use large language models in medicine.

Approach	Requirements	Pros	Cons	Examples
**Few-shot learning**	Several exemplars	1. Dealing with edge cases; 2. Specifying expected styles	Exemplars might introduce biases	MedPrompt^13^
**Tool learning**	Application programming interfaces	Providing domain functionalities	Relies on the curation of tools	GeneGPT^65^, EHRAgent^66^, ChemCrow^67^
**Chain-of-thought prompting**	Additional prompt text (“Let’s think step-by-step.”)	1. Providing explanations; 2. Improving performance	Hard to parse (mitigated by structured output)	MedPrompt^13^
**Retrieval-augmented generation**	A knowledge base or document collection	1. Providing up-to-date knowledge; 2. Reducing hallucinations	Depends on the quality of the retrieved documents	Almanac^68^, MedRAG^54^
**Fine-tuning**	Data annotations and compute	1. Improving performance 2. Shorten the prompt	Costly and resource intensive	MEDITRON^52^, PMC-LlaMA^51^

**Table 3.** Representative case studies of utilizing large language models in medicine.

Study	Task Formulation	LLM(s) Selection	Technique	Evaluation
Van Veen et al.^20^	Summarization	FLAN-T5^75^, FLAN-UL2^76^, Alpaca^77^, Med-Alpaca^78^, Vicuna^79^, Llama-2^4^	Few-shot learning, fine-tuning	Automatic and manual evaluation of clinical summarization
Singhal et al.^14^	Knowledge and Reasoning	PaLM^80^, Flan-PaLM^75^	Few-shot learning, chain-of-thought prompting, fine-tuning	MCQ evaluation and manual evaluation of question answering
Wang et al.^44^	Structurization	Llama^4^, ClinicalBERT^81^	Fine-tuning	Automatic classification evaluation and manual error analysis
Mirza et al.^43^	Translation	GPT-4^1^	Direct prompting	Manual evaluation of clinical translation by clinicians and legal experts
Zhanget al.^82^	Multi-modality	BiomedGPT^82^	Fine-tuning	MCQ evaluation and manual evaluation of visual tasks

## Box 1. Best Practices

1. Organize your task into one of the five categories: (1) **knowledge and reasoning**, (2) **summarization**, (3) **translation**, (4) **structurization**, and (5) **multi-modal data analysis**. Collect up to 100 test cases for evaluation with task-specific metrics. (Figure 2)
2. Ensure that the LLM usage is compliant to Health Insurance Portability and Accountability Act (**HIPAA**) if working with sensitive data.
3. Choose an LLM that can process the **data modality** and has sufficient **context length** (length of the input prompt) used in the task. (Figure 3)
4. Select LLMs based on task-specific performance evaluated by experts in the literature. Scores on medical examinations can be used for the initial screening of LLMs (Figure 3)
5. Use one to five representative and diverse examples in **few-shot learning** to better specify the response style and handle edge cases. (Figure 4)
6. Always ask LLMs to “think-step-by-step” and generate the rationale before the answer for better explainability and improved performance. (**chain-of-thought prompting,** Figure 4)
7. Use **retrieval-augmented generation (RAG)** or tool learning if knowledge or domain utilities are needed and to make evidence-based and up-to-date generation. (Figure 4)
8. Use a **temperature** of 0 and **JSON formatting** to generate reproducible and structured outputs that can be easily parsed. (Figure 4)
9. Consider **fine-tuning** when prompt engineering fails to reach the desired performance, the working prompt is too costly, or abundant training data is readily available. (Figure 4)
10. Safeguard against potential risks by monitoring **fairness**, **equity**, **bias**, and **cost** when deploying LLMs in the biomedical domain.

## Box 2. Glossary

1. **Large language model:** A large language model is a type of artificial intelligence designed to process and generate human-like text. The model is built using deep learning techniques and is trained on text corpora to perform tasks such as language translation, summarization, and question answering.
2. **Instance:** An instance refers to a specific example used for training or testing a model. Each instance typically includes input data and corresponding output, which the model is expected to predict or generate.
3. **Rationale:** Rationale is text output produced by a language model that provides additional context or reasoning for an output answer or value.
4. **Multi-modal comprehension**: Refers to the ability of models like GPT-4 to analyze and integrate diverse data types, including text, images, audio, and genomic information, for various applications such as medicine and research.
5. **Parameter:** A parameter is a variable within a large language model that is learned from training data and used to make predictions. The value of a parameter is often adjusted to minimize error during the training phase.
6. **Context Window:** Context window refers to the number of tokens that a language model can receive as input. Larger context windows can increase the ability to perform in-context learning within a large language model prompt.
7. **Token**: A token is the smallest unit of information that a language model can process. Tokens often represent text information like single letters, spaces, sub-words, words, or phrases. According to empirical evidence, one token is about 0.75 of a word.
8. **Prompt engineering**: Prompt engineering involves crafting inputs or “prompts” that guide large language models to generate desired outputs without changing their parameters. Effective prompt design can significantly enhance the performance of LLMs.
9. **Few-shot learning**: Few-shot learning refers to the ability of a model to learn a new task from a very limited number of examples (shots). In the context of LLMs, this involves providing a few specific examples during the prompt to guide the model on how to handle similar tasks, improving its accuracy and adaptability.
10. **Retrieval-augmented generation**: Retrieval-augmented generation combines traditional language modeling with a retrieval component that searches for relevant information. This enhances the model’s responses by grounding them in factual data.
11. **Hallucination**: Hallucination refers to instances where the model generates incorrect or fabricated information.
12. **Chain-of-thought prompting**: Chain-of-thought prompting is a technique used with large language models to encourage step-by-step reasoning in their responses. By explicitly asking the model to think through the steps of a problem, it can provide more transparent and logically structured solutions.
13. **Temperature**: Temperature controls the randomness of the generated responses from large language models. A higher temperature increases diversity in the output, leading to more creative and varied responses, while a lower temperature produces more predictable and consistent outputs.
14. **Fine-tuning**: Fine-tuning is an approach to transfer learning in which pre-trained model parameters are further modified on a new dataset.
15. **HIPAA-compliance**: HIPAA-compliant systems adhere to HIPAA (Health Insurance Portability and Accountability Act) regulations, ensuring they meet the required standards to protect patient health information. HIPAA sets national standards for the protection of health information in the United States. It ensures the privacy and security of individually identifiable health information.

## References

[1] Achiam J., Gpt-4 technical report. arXiv preprint arXiv:2303.08774 (2023).

[2] Anthropic A. The claude 3 model family: Opus, sonnet, haiku. Claude-3 Model Card (2024).

[3] Reid M. Gemini 1.5: Unlocking multimodal understanding across millions of tokens of context. arXiv preprint arXiv:2403.05530 (2024).

[4] The llama 3 herd of models. arXiv preprint arXiv:2407.21783 (2024.

[5] Brown T., Language models are few-shot learners. Adv. Neural Inf. Process. Syst. 33, 1877–1901 (2020).

[6] Ouyang L. Training language models to follow instructions with human feedback. Adv. Neural Inf. Process. Syst. 35, 27730–27744 (2022).

[7] Mukherjee P., Hou B., Lanfredi R.B. & Summers R.M. Feasibility of Using the Privacy-preserving Large Language Model Vicuna for Labeling Radiology Reports. Radiology 309, e231147 (2023). 37815442 10.1148/radiol.231147 PMC10623189

[8] Tian S., Opportunities and challenges for ChatGPT and large language models in biomedicine and health. Brief Bioinform 25 (2023). 10.1093/bib/bbad493 PMC10762511 38168838

[9] Thirunavukarasu A.J., Large language models in medicine. Nat. Med. 29, 1930–1940 (2023). 37460753 10.1038/s41591-023-02448-8

[10] Nori H., King N., McKinney S.M., Carignan D. & Horvitz E. Capabilities of gpt-4 on medical challenge problems. arXiv preprint arXiv:2303.13375 (2023).

[11] Saab K., Capabilities of gemini models in medicine. arXiv preprint arXiv:2404.18416 (2024).

[12] Liévin V., Hother C.E., Motzfeldt A.G. & Winther O. Can large language models reason about medical questions? Patterns (N. Y.) 5, 100943 (2024). 38487804 10.1016/j.patter.2024.100943 PMC10935498

[13] Nori H., Can generalist foundation models outcompete special-purpose tuning? case study in medicine. arXiv preprint arXiv:2311.16452 (2023).

[14] Singhal K., Large language models encode clinical knowledge. Nature 620, 172–180 (2023). 37438534 10.1038/s41586-023-06291-2 PMC10396962

[15] Hu X. Interpretable medical image visual question answering via multi-modal relationship graph learning. Med. Image Anal. 97, 103279 (2024). 39079429 10.1016/j.media.2024.103279

[16] Jin Q., Matching Patients to Clinical Trials with Large Language Models. arXiv (2024). 10.1038/s41467-024-53081-z PMC11574183 39557832

[17] Wong C., Scaling clinical trial matching using large language models: A case study in oncology. Machine Learning for Healthcare Conference 846–862 (PMLR, 2023).

[18] Wornow M., Zero-Shot Clinical Trial Patient Matching with LLMs. arXiv preprint arXiv:2402.05125 (2024).

[19] Roberts K. Large language models for reducing clinicians’ documentation burden. Nat. Med. 30, 942–943 (2024). 38561439 10.1038/s41591-024-02888-w

[20] Van Veen D., Adapted large language models can outperform medical experts in clinical text summarization. Nat. Med. 1–9 (2024). 38413730 10.1038/s41591-024-02855-5 PMC11479659

[21] Patel S.B. & Lam K. ChatGPT: the future of discharge summaries? Lancet Digit. Health 5, e107–e108 (2023). 36754724 10.1016/S2589-7500(23)00021-3

[22] Moor M., Foundation models for generalist medical artificial intelligence. Nature 616, 259–265 (2023). 37045921 10.1038/s41586-023-05881-4

[23] Acosta J.N., Falcone G.J., Rajpurkar P. & Topol E.J. Multimodal biomedical AI. Nat. Med. 28, 1773–1784 (2022). 36109635 10.1038/s41591-022-01981-2

[24] Omiye J. A., Gui H., Rezaei S. J., Zou J. & Daneshjou R. Large language models in medicine: the potentials and pitfalls: a narrative review. Ann. Intern. Med. 177, 210–220 (2024). 38285984 10.7326/M23-2772

[25] Hu Y. Improving large language models for clinical named entity recognition via prompt engineering. J. Am. Med. Inform. Assoc. 31, 1812–1820 (2024). 38281112 10.1093/jamia/ocad259 PMC11339492

[26] Wang L. Prompt engineering in consistency and reliability with the evidence-based guideline for LLMs. NPJ Digit. Med 7, 41 (2024). 38378899 10.1038/s41746-024-01029-4 PMC10879172

[27] Jin Q. Hidden flaws behind expert-level accuracy of multimodal GPT-4 vision in medicine. NPJ Digit Med 7, 190 (2024). 39043988 10.1038/s41746-024-01185-7 PMC11266508

[28] Gilson A., How Does ChatGPT Perform on the United States Medical Licensing Examination (USMLE)? The Implications of Large Language Models for Medical Education and Knowledge Assessment. JMIR Med. Educ. 9, e45312 (2023). 36753318 10.2196/45312 PMC9947764

[29] Singhal K., Towards expert-level medical question answering with large language models. arXiv preprint arXiv:2305.09617 (2023). 10.1038/s41591-024-03423-7 PMC11922739 39779926

[30] Jin Q., Leaman R. & Lu Z. Retrieve, Summarize, and Verify: How Will ChatGPT Affect Information Seeking from the Medical Literature? J. Am. Soc. Nephrol. 34, 1302–1304 (2023). 37254254 10.1681/ASN.0000000000000166 PMC10400098

[31] Liu S., Using AI-generated suggestions from ChatGPT to optimize clinical decision support. J. Am. Med. Inform. Assoc. 30, 1237–1245 (2023). 37087108 10.1093/jamia/ocad072 PMC10280357

[32] Tang L., Evaluating large language models on medical evidence summarization. NPJ Digit. Med. 6, 158 (2023). 37620423 10.1038/s41746-023-00896-7 PMC10449915

[33] Shaib C., Summarizing, simplifying, and synthesizing medical evidence using GPT-3 (with varying success). arXiv preprint arXiv:2305.06299 (2023). 10.18653/v1/2023.acl-short.119 PMC11613457 39629494

[34] Zhang G. Closing the gap between open source and commercial large language models for medical evidence summarization. NPJ Digit. Med 7, 239 (2024). 39251804 10.1038/s41746-024-01239-w PMC11383939

[35] Peng Y., Rousseau J.F., Shortliffe E.H. & Weng C. AI-generated text may have a role in evidence-based medicine. Nat. Med. 29, 1593–1594 (2023). 37221382 10.1038/s41591-023-02366-9 PMC11193148

[36] Papineni K., Roukos S., Ward T. & Zhu W.-J. Bleu: a method for automatic evaluation of machine translation. Proc. Assoc. Comput. Linguist. 311–318 (2002).

[37] Lin C.-Y. Rouge: A package for automatic evaluation of summaries. in Text summarization branches out 74–81 (2004).

[38] Zhang T., Kishore V., Wu F., Weinberger K.Q. & Artzi Y. BERTScore: Evaluating Text Generation with BERT. International Conference on Learning Representations (2019).

[39] Wang L.L., Automated Metrics for Medical Multi-Document Summarization Disagree with Human Evaluations. Proc. Assoc. Comput. Linguist. 9871–9889 (2023). 10.18653/v1/2023.acl-long.549 PMC11613456 39629493

[40] Team NLLB. Scaling neural machine translation to 200 languages. Nature 1–6 (2024). 10.1038/s41586-024-07335-x PMC11208141 38839963

[41] Waisberg E., Ong J., Masalkhi M. & Lee A.G. Large language model (LLM)-driven chatbots for neuro-ophthalmic medical education. Eye 38, 639–641 (2024). 37749374 10.1038/s41433-023-02759-7 PMC10920622

[42] Eysenbach G. The role of ChatGPT, generative language models, and artificial intelligence in medical education: a conversation with ChatGPT and a call for papers. JMIR Med. Educ. 9, e46885 (2023). 36863937 10.2196/46885 PMC10028514

[43] Mirza F.N., Using chatgpt to facilitate truly informed medical consent. NEJM AI 1, AIcs2300145 (2024).

[44] Wang H., Gao C., Dantona C., Hull B. & Sun J. DRG-LLaMA: tuning LLaMA model to predict diagnosis-related group for hospitalized patients. NPJ Digit. Med. 7, 16 (2024). 38253711 10.1038/s41746-023-00989-3 PMC10803802

[45] Dagdelen J., Structured information extraction from scientific text with large language models. Nat. Commun. 15, 1418 (2024). 38360817 10.1038/s41467-024-45563-x PMC10869356

[46] Topol E.J. As artificial intelligence goes multimodal, medical applications multiply. Science 381, adk6139 (2023). 37708283 10.1126/science.adk6139

[47] Chen P.C., Liu Y. & Peng L. How to develop machine learning models for healthcare. Nat. Mater. 18, 410–414 (2019). 31000806 10.1038/s41563-019-0345-0

[48] Doshi R., Quantitative Evaluation of Large Language Models to Streamline Radiology Report Impressions: A Multimodal Retrospective Analysis. Radiology 310, e231593 (2024). 38530171 10.1148/radiol.231593

[49] Jiang A.Q. Mixtral of experts. arXiv preprint arXiv:2401.04088 (2024).

[50] Minaee S. Large language models: A survey. arXiv preprint arXiv:2402.06196 (2024).

[51] Wu C. PMC-LLaMA: toward building open-source language models for medicine. J Am Med Inform Assoc 31, 1833–1843 (2024). 38613821 10.1093/jamia/ocae045 PMC11639126

[52] Chen Z. Meditron-70b: Scaling medical pretraining for large language models. arXiv preprint arXiv:2311.16079 (2023).

[53] Jin D. What Disease Does This Patient Have? A large-scale open domain question answering dataset from medical exams. Appl. Sci. 11, 6421 (2021).

[54] Xiong G., Jin Q., Lu Z. & Zhang A. Benchmarking retrieval-augmented generation for medicine. Findings of the Association for Computational Linguistics: ACL 6233–6251 (2024).

[55] Shi W. MedAdapter: Efficient Test-Time Adaptation of Large Language Models towards Medical Reasoning. arXiv preprint arXiv:2405.03000 (2024). 10.18653/v1/2024.emnlp-main.1244 PMC11868705 40028445

[56] Pal A. & Sankarasubbu M. Gemini goes to med school: exploring the capabilities of multimodal large language models on medical challenge problems & hallucinations. arXiv preprint arXiv:2402.07023 (2024).

[57] University Stanford. Holistic Evaluation of Language Models (HELM). Available at: https://nlp.stanford.edu/helm/v2-lite-finch/?group=med_qa (2024).

[58] Cui H. scGPT: toward building a foundation model for single-cell multi-omics using generative AI. Nat. Methods 21, 1470–1480 (2024). 38409223 10.1038/s41592-024-02201-0

[59] Liu N. F. Lost in the middle: How language models use long contexts. Trans. Assoc. Comput. Linguist. 12, 157–173 (2024).

[60] Devlin J., Chang M.-W., Lee K. & Toutanova K. BERT: Pre-training of Deep Bidirectional Transformers for Language Understanding. Assoc. Comput. Linguist. 4171–4186 (2019).

[61] Chen Q. Large language models in biomedical natural language processing: benchmarks, baselines, and recommendations. arXiv preprint arXiv:2305.16326 (2023).

[62] Jin Q. PubMedQA: A Dataset for Biomedical Research Question Answering. Proceedings of the 2019 Conference on Empirical Methods in Natural Language Processing and the 9th International Joint Conference on Natural Language Processing (EMNLP-IJCNLP) (2019).

[63] Pal A., Umapathi L. K. & Sankarasubbu M. Medmcqa: A large-scale multi-subject multi-choice dataset for medical domain question answering. Conference on Health, Inference, and Learning (PMLR, 2022).

[64] Liu P., Pre-train, prompt, and predict: A systematic survey of prompting methods in natural language processing. ACM Comput. Surv. 55, 1–35 (2023).

[65] Jin Q., Yang Y., Chen Q. & Lu Z. GeneGPT: augmenting large language models with domain tools for improved access to biomedical information. Bioinformatics 40 (2024). 10.1093/bioinformatics/btae075 PMC10904143 38341654

[66] Shi W. EHRAgent: Code Empowers Large Language Models for Few-shot Complex Tabular Reasoning on Electronic Health Records. in ICLR 2024 Workshop on Large Language Model (LLM) Agents. 10.18653/v1/2024.emnlp-main.1245 PMC11867733 40018366

[67] M Bran A. Augmenting large language models with chemistry tools. Nat Mach Intell 6, 525–535 (2024). 38799228 10.1038/s42256-024-00832-8 PMC11116106

[68] Zakka C. Almanac - Retrieval-Augmented Language Models for Clinical Medicine. NEJM AI 1 (2024). 10.1056/aioa2300068 PMC10857783 38343631

[69] Wei J., Chain-of-thought prompting elicits reasoning in large language models. Adv. Neural Inf. Process. Syst. 35, 24824–24837 (2022).

[70] Jin Q. Biomedical question answering: a survey of approaches and challenges. ACM Comput. Surv. 55, 1–36 (2022).

[71] Ji Z. Survey of hallucination in natural language generation. ACM Computing Surveys 55, 1–38 (2023).

[72] Wang X. Self-consistency improves chain of thought reasoning in language models. arXiv preprint arXiv:2203.11171 (2022).

[73] Hou W. & Ji Z. Assessing GPT-4 for cell type annotation in single-cell RNA-seq analysis. Nat. Methods (2024). 10.1038/s41592-024-02235-4 PMC11310073 38528186

[74] Cosentino Justin, “Towards a Personal Health Large Language Model.” arXiv preprint arXiv:2406.06474 (2024).

[75] Chung H.W., Scaling instruction-finetuned language models. J. Mach. Learn. Res. 25, 1–53 (2024).

[76] Tay Y. Ul2: Unifying language learning paradigms. arXiv preprint arXiv:2205.05131 (2022).

[77] Stanford ALPACA team. ALPACA. Available at: https://github.com/tatsu-lab/stanford_alpaca. (2024)

[78] Han T. MedAlpaca--an open-source collection of medical conversational AI models and training data. arXiv preprint arXiv:2304.08247 (2023).

[79] Chiang W.-L. Vicuna: An open-source chatbot impressing gpt-4 with 90%* chatgpt quality. Available at: https://vicuna.lmsys.org (2023).

[80] Chowdhery A. Palm: Scaling language modeling with pathways. J. Mach. Learn. Res. 24, 1–113 (2023).

[81] Alsentzer E. Publicly available clinical BERT embeddings. Proceedings of the 2nd Clinical Natural Language Processing Workshop. 72–78 (2019).

[82] Zhang K. A generalist vision-language foundation model for diverse biomedical tasks. Nat. Med. (2024). 10.1038/s41591-024-03185-2 PMC12581140 39112796

[83] Hu E.J., LoRA: Low-Rank Adaptation of Large Language Models. International Conference on Learning Representations (2021).

[84] Dettmers T., Qlora: Efficient finetuning of quantized llms. Adv. Neural Inf. Process. Syst. 36 (2024).

[85] Li L., Li Q., Zhang B. & Chu X. Norm tweaking: high-performance low-bit quantization of large language models. Proc. AAAI Conf. Artif. Intell. 38, 18536–18544 (2024).

[86] Lialin V., Deshpande V. & Rumshisky A. Scaling down to scale up: A guide to parameter-efficient fine-tuning. arXiv preprint arXiv:2303.15647 (2023).

[87] Biderman D. Lora learns less and forgets less. arXiv preprint arXiv:2405.09673 (2024).

[88] Kuzmin A., Fp8 quantization: The power of the exponent. Adv. Neural Inf. Process. Syst. 35, 14651–14662 (2022).

[89] Van Veen D. Adapted large language models can outperform medical experts in clinical text summarization. Nat Med 30, 1134–1142 (2024). 38413730 10.1038/s41591-024-02855-5 PMC11479659

[90] General Data Protection Regulation (GDPR) – Legal Text. General Data Protection Regulation (GDPR). Available at https://gdpr-info.eu/. (2024)

[91] Yang Y., A survey of recent methods for addressing AI fairness and bias in biomedicine. J. Biomed. Inform. 104646 (2024). 10.1016/j.jbi.2024.104646 PMC11129918 38677633

[92] Omiye J.A., Lester J.C., Spichak S., Rotemberg V. & Daneshjou R. Large language models propagate race-based medicine. NPJ Digit. Med. 6, 195 (2023). 37864012 10.1038/s41746-023-00939-z PMC10589311

[93] Zhang G. Leveraging generative AI for clinical evidence synthesis needs to ensure trustworthiness. J Biomed Inform 153, 104640 (2024). 38608915 10.1016/j.jbi.2024.104640 PMC11217921

[94] Sun L. Trustllm: Trustworthiness in large language models. arXiv preprint arXiv:2401.05561 (2024).

[95] Wang B., “DecodingTrust: A Comprehensive Assessment of Trustworthiness in GPT Models.” Adv. Neural Inf. Process. Syst. (2023).

[96] Meskó B. & Topol E.J. The imperative for regulatory oversight of large language models (or generative AI) in healthcare. NPJ Digit. Med. 6, 120 (2023). 37414860 10.1038/s41746-023-00873-0 PMC10326069

[97] Clark C.R., Health Care Equity in the Use of Advanced Analytics and Artificial Intelligence Technologies in Primary Care. J. Gen. Intern. Med. 36, 3188–3193 (2021). 34027610 10.1007/s11606-021-06846-x PMC8481410

